# Proximal and distal deep venous thrombosis in critically ill patients: incidence and prevalence

**DOI:** 10.1186/cc11025

**Published:** 2012-03-20

**Authors:** S Yus Teruel, J Camacho Oviedo, L Cachafeiro Fuciños, M Hernandez Bernal, A Agrifoglio Rotaeche, M Irazábal Jaimes, L Fernandez Rodriguez, B Civantos Martín, J Díez

**Affiliations:** 1 Hospital Universitario La Paz, Madrid, Spain

## Introduction

The aim of this study was to detect deep venous thrombosis (DVT) in patients admitted to a critical care unit (ICU) by compression ultrasonography, and to determine the incidence and prevalence of proximal and distal DVT in this setting.

## Methods

This was a prospective observational study conducted in our medical-surgical and trauma ICU from October 2009 to September 2010. The inclusion criterion was ≥72 hours of ICU stay. Exclusion criteria were admitting diagnosis of pulmonary embolism or DVT, readmission, and patients with support withdrawal orders. The study was approved by the Research Ethics Board of La Paz Hospital. Bilateral lower extremity compression ultrasound was performed within 48 hours of admission to evaluate the prevalence, and twice weekly until discharge to assess the incidence. We collected demographic data, body mass index (BMI), APACHE II score, SOFA score, diagnostic categories, classic risk factors for DVT, femoral catheter and the use of mechanical ventilation and muscle relaxants. For the statistical analysis chi-square and Fisher tests were used, as well as Mann-Whitney and Student tests for data comparison. For the probability of DVT and its relation with the associated factors, the odds ratio and confidence interval were used. Statistical significance was *P *< 0.05.

## Results

We enrolled 182 patients, with male predominance (57.3%), 135 were mechanically ventilated (74.2%) and the mean APACHE II score was 19.3 ( ± 7.8). The mortality in the ICU was 15.4% (28), and 20.9 (38) in hospital. The prevalence of proximal DVT was 29.1% (53/182), and the incidence 24.0% (31/129). Seventy-nine percent of patients received DVT prophylaxis. The localization of incidentally diagnosed DVT was proximal in 29% and distal in 35%; 19 (64%) of these were identified on day 5 of admission. In four patients DVT was clinically suspected and only in one of them was DVT confirmed. The most frequently involved were soleal veins (67%). Independent risk factors for incidental DVT were: older age (62 ± 15.4 years vs. 54.5 ± 17.1; *P *= 0.032); BMI (27.7 ± 5.5 kg/m^2 ^vs. 24.9 ± 5.2 kg/m^2^; *P *= 0.014); and mechanical ventilation: (OR: 3.3, 95% CI = 1.0 to 10.26). Patients with incidental DVT had a higher hospital mortality (*P *= 0.03).

## Conclusion

In our study DVT was an early, asymptomatic and frequent event (46% of the ICU patients). In the presence of risk factors, a diagnostic ultrasound test might have a role.

